# 5-Hydr­oxy-1-methyl-3,4-dihydro-2*H*-pyrrolium hydrogensulfate

**DOI:** 10.1107/S1600536808022460

**Published:** 2008-07-31

**Authors:** Yan-Xiong Fang, Hua-Rong Huang, Zhi-Yun Du, Bao-Hua Huang, Kun Zhang

**Affiliations:** a Faculty of Light Industrial and Chemical Engineering, Guangdong University of Technology, Guangzhou 510090, People’s Republic of China

## Abstract

The title compound, C_5_H_10_NO^+^·HSO_4_^−^, has been synthesized by reaction of 1-methyl­pyrrolidin-2-one with H_2_SO_4_ in a 1:1 molar ratio. The substituted pyrrolium ring adopts an envelope conformation. The hydrogensulfate anions form infinite helical chains parallel to the *a* axis *via* strong O—H⋯O hydrogen bonds. The pyrrolium cations are pendant from the chains. These cations are the hydrogen donors in the strong O—H⋯O hydrogen bonds to the hydrogensulfates. In addition, there are weak C—H⋯O hydrogen bonds in the structure.

## Related literature

For related literature, see: Forbes & Weaver (2004 ▶); Zhu *et al.* (2003 ▶); Desiraju & Steiner (1999 ▶).
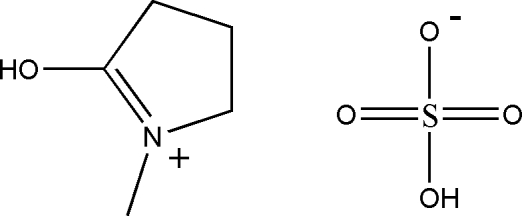


## Experimental

### 

#### Crystal data

C_5_H_10_NO^+^·HSO_4_^−^
*M* *_r_* = 197.21
Orthorhombic, 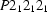

*a* = 6.5418 (14) Å
*b* = 10.964 (2) Å
*c* = 11.614 (2) Å
*V* = 833.0 (3) Å^3^
*Z* = 4
Mo *K*α radiation
μ = 0.37 mm^−1^
*T* = 173 (2) K
0.48 × 0.25 × 0.22 mm

#### Data collection

Bruker SMART 1K area-detector diffractometer
Absorption correction: multi-scan (*SADABS*; Sheldrick, 1996 ▶) *T*_min_ = 0.841, *T*_max_ = 0.922
4132 measured reflections
1578 independent reflections
1519 reflections with *I* > 2σ(*I*)
*R*_int_ = 0.023

#### Refinement

*R*[*F* ^2^ > 2σ(*F* ^2^)] = 0.027
*wR*(*F* ^2^) = 0.089
*S* = 1.20
1578 reflections
113 parameters
H-atom parameters constrained
Δρ_max_ = 0.28 e Å^−3^
Δρ_min_ = −0.36 e Å^−3^
Absolute structure: Flack (1983 ▶), 609 Friedel pairs
Flack parameter: 0.01 (9)

### 

Data collection: *SMART* (Bruker, 1999 ▶); cell refinement: *SAINT-Plus* (Bruker, 1999 ▶); data reduction: *SAINT-Plus*; program(s) used to solve structure: *SHELXS97* (Sheldrick, 2008 ▶); program(s) used to refine structure: *SHELXL97* (Sheldrick, 2008 ▶); molecular graphics: *SHELXTL* (Sheldrick, 2008 ▶); software used to prepare material for publication: *SHELXTL*.

## Supplementary Material

Crystal structure: contains datablocks I, global. DOI: 10.1107/S1600536808022460/fb2100sup1.cif

Structure factors: contains datablocks I. DOI: 10.1107/S1600536808022460/fb2100Isup2.hkl

Additional supplementary materials:  crystallographic information; 3D view; checkCIF report

## Figures and Tables

**Table 1:** Hydrogen-bond geometry (Å, °)

*D*—H⋯*A*	*D*—H	H⋯*A*	*D*⋯*A*	*D*—H⋯*A*
O3—H3⋯O5^i^	0.84	1.75	2.569 (3)	164
O1—H1⋯O2	0.84	1.70	2.540 (2)	177
C2—H2*A*⋯O5^ii^	0.99	2.45	3.250 (3)	137
C5—H5*C*⋯O2^iii^	0.98	2.59	3.488 (3)	152

Symmetry codes: (i) 
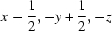
; (ii) 
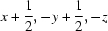
; (iii) 
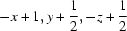
.

**Fig. 1.**
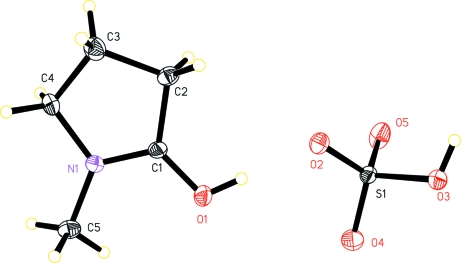
The molecules in the asymmetric unit of the title compound, with anisotropic displacement parameters drawn at the 50% probability level.

**Fig. 2.**
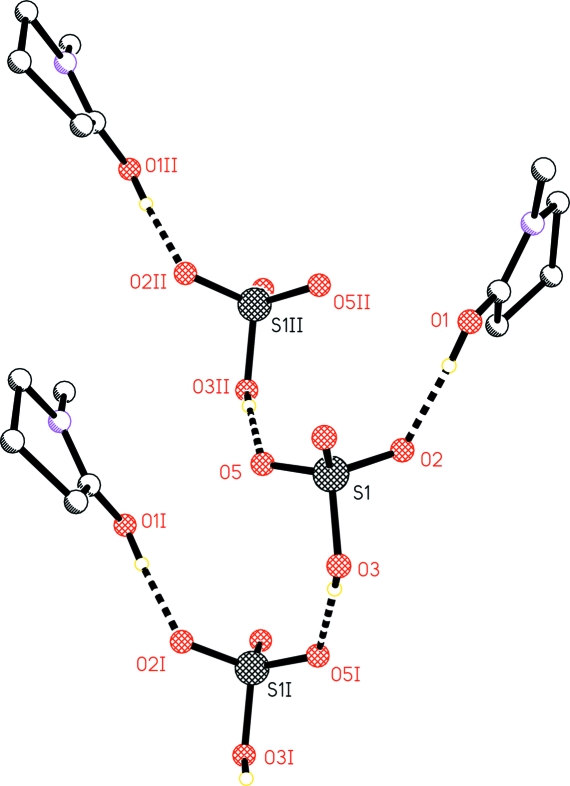
A view of the O—H···O hydrogen-bond pattern. The H atoms that are not involved in the O—H···O hydrogen bonds have been omitted for the sake of clarity. The chains of the hydrogensulfates are oriented parallel to the crystallographic axis a. Symmetry codes: (I) x - 1/2,0.5 - y,-z; (II) x + 1/2,0.5 - y,-z.

## supplementary crystallographic information

### Comment

1-methyl-2-hydroxyl-pyrrolium hydrogensulfate is applied in the green chemical engineering field as a replacement of volatile organic solvents (Forbes *et al.*, 2004) or as a catalyst for esterification (Zhu *et al.*, 2003).

In the title structure, the bond distances and angles are normal. The most important structural feature is presence of strong intermolecular O—H···O hydrogen bonds (Desiraju & Steiner, 1999) that interconnect the hydrogensulfate anions (Tab. 1). The hydrogensulfates form infinite left-handed helical chains along the axis *a*. The hydrogensulfates are acceptors of another short hydrogen O—H···O bond donated by the 1-methyl-2-hydroxyl-pyrrolium cations (Tab. 1). In addition, there are also C—H···O weak hydrogen bonds present in the structure (Tab. 1).

The unconstrained refinement of the hydroxyl hydrogens resulted in the less probable distances: 0.92 (4) and 0.72 (4)Å for O1-H1 and O3-H3, respectively.

### Experimental

The title compound was prepared by the reaction of 1-methylpyrrolidin-2-one and H_2_SO_4_ in 1:1 mole ratio. 3.675 g (0.0375 mol) H_2_SO_4_ was added dropwise under stirring at room temperature to a boiling flask containing 3.712 g (0.0375 mol) of 1-methylpyrrolidin-2-one. Then the mixture was heated to 373 K. After 2 h, the mixture was cooled to room temperature and the title compound was obtained. Its crystals of were obtained from petroleum/ethyl acetate (*v*/*v* = 1/1) by solvent evaporation at 4° C. The longest dimension of the crystals was about 10 mm. The compound's identity was confirmed by IR and NMR spectra. ^1^H NMR in CD_3_CN (500 MHz): 5.4–6.3(H),3.59 (t, 7 Hz, 2H), 2.94 (s, 3H), 2.74(t, 8 Hz, 2H), 2.10 (m, 8 Hz, 2H).

### Refinement

All the H atoms were discernible in the difference Fourier maps. However, the H atoms were constrained in a riding-motion approximation. *C*—H_methyl_ 0.98, *C*—H_methylene_0.99, O—H 0.84 Å. *U*_iso_(H_methylene_)=1.2*U*_eq_(C_methylene_); *U*_iso_(H_methyl_)=1.5*U*_eq_(C_methyl_); *U*_iso_(H_O_)=1.5(O).

### Crystal data

**Table:**

C_5_H_10_NO^+^·HSO_4_^–^	*F*_000_ = 416
*M**_r_* = 197.21	*D*_x_ = 1.573 Mg m^−^^3^
Orthorhombic, *P*2_1_2_1_2_1_	Mo *K*α radiation λ = 0.71073 Å
Hall symbol: P 2ac 2ab	Cell parameters from 3942 reflections
*a* = 6.5418 (14) Å	θ = 2.6–27.0º
*b* = 10.964 (2) Å	µ = 0.37 mm^−^^1^
*c* = 11.614 (2) Å	*T* = 173 (2) K
*V* = 833.0 (3) Å^3^	Prism, colourless
*Z* = 4	0.48 × 0.25 × 0.22 mm

### Data collection

**Table:**

Bruker SMART 1K area-detector diffractometer	1578 independent reflections
Radiation source: medium-focus sealed tube	1519 reflections with *I* > 2σ(*I*)
Monochromator: graphite	*R*_int_ = 0.023
*T* = 173(2) K	θ_max_ = 26.0º
φ and ω scans	θ_min_ = 2.6º
Absorption correction: multi-scan(SADABS; Sheldrick, 1996)	*h* = −7→8
*T*_min_ = 0.841, *T*_max_ = 0.922	*k* = −13→12
4132 measured reflections	*l* = −10→14

### Refinement

**Table:**

Refinement on *F*^2^	Hydrogen site location: difference Fourier map
Least-squares matrix: full	H-atom parameters constrained
*R*[*F*^2^ > 2σ(*F*^2^)] = 0.027	*w* = 1/[σ^2^(*F*_o_^2^) + (0.047*P*)^2^ + 0.264*P*] where *P* = (*F*_o_^2^ + 2*F*_c_^2^)/3
*wR*(*F*^2^) = 0.089	(Δ/σ)_max_ < 0.001
*S* = 1.20	Δρ_max_ = 0.28 e Å^−^^3^
1578 reflections	Δρ_min_ = −0.36 e Å^−^^3^
113 parameters	Extinction correction: SHELXL97 (Sheldrick, 2008), Fc^*^=kFc[1+0.001xFc^2^λ^3^/sin(2θ)]^-1/4^
41 constraints	Extinction coefficient: 0.020 (4)
Primary atom site location: structure-invariant direct methods	Absolute structure: Flack (1983)
Secondary atom site location: difference Fourier map	Flack parameter: 0.01 (9)

### Special details

**Table:**

Geometry. All e.s.d.'s (except the e.s.d. in the dihedral angle between two l.s. planes) are estimated using the full covariance matrix. The cell e.s.d.'s are taken into account individually in the estimation of e.s.d.'s in distances, angles and torsion angles; correlations between e.s.d.'s in cell parameters are only used when they are defined by crystal symmetry. An approximate (isotropic) treatment of cell e.s.d.'s is used for estimating e.s.d.'s involving l.s. planes.
Refinement. Refinement of *F*^2^ against ALL reflections. The weighted *R*-factor *wR* and goodness of fit *S* are based on *F*^2^, conventional *R*-factors *R* are based on *F*, with *F* set to zero for negative *F*^2^. The threshold expression of *F*^2^ > σ(*F*^2^) is used only for calculating *R*-factors(gt) *etc*. and is not relevant to the choice of reflections for refinement. *R*-factors based on *F*^2^ are statistically about twice as large as those based on *F*, and *R*- factors based on ALL data will be even larger.

### Fractional atomic coordinates and isotropic or equivalent isotropic displacement parameters (Å^2^)

**Table:**

	*x*	*y*	*z*	*U*_iso_*/*U*_eq_	
C1	0.6096 (4)	0.42511 (19)	0.26605 (17)	0.0238 (5)	
C2	0.5508 (4)	0.3055 (2)	0.31637 (19)	0.0275 (5)	
H2A	0.5667	0.2389	0.2596	0.033*	
H2B	0.4078	0.3068	0.3443	0.033*	
C3	0.7017 (4)	0.2912 (2)	0.4163 (2)	0.0346 (6)	
H3A	0.6369	0.3142	0.4902	0.042*	
H3B	0.7507	0.2060	0.4219	0.042*	
C4	0.8773 (4)	0.3774 (2)	0.38749 (19)	0.0296 (5)	
H4A	0.9259	0.4207	0.4571	0.035*	
H4B	0.9932	0.3326	0.3526	0.035*	
C5	0.8878 (4)	0.5749 (2)	0.2701 (2)	0.0309 (5)	
H5A	0.8521	0.5941	0.1902	0.046*	
H5B	1.0361	0.5644	0.2766	0.046*	
H5C	0.8438	0.6418	0.3203	0.046*	
N1	0.7857 (3)	0.46269 (17)	0.30466 (16)	0.0242 (4)	
O1	0.5090 (3)	0.48791 (14)	0.19136 (14)	0.0299 (4)	
H1	0.4010	0.4508	0.1739	0.045*	
O5	0.1546 (3)	0.27995 (19)	−0.05492 (17)	0.0434 (5)	
O3	−0.1165 (2)	0.40674 (14)	0.01936 (15)	0.0318 (4)	
H3	−0.1724	0.3407	0.0382	0.048*	
O4	0.1944 (3)	0.49886 (18)	−0.03734 (17)	0.0418 (5)	
O2	0.1898 (3)	0.37202 (16)	0.13185 (14)	0.0308 (4)	
S1	0.11940 (8)	0.38873 (5)	0.01336 (4)	0.02365 (18)	

### Atomic displacement parameters (Å^2^)

**Table:**

	*U*^11^	*U*^22^	*U*^33^	*U*^12^	*U*^13^	*U*^23^
C1	0.0289 (12)	0.0234 (10)	0.0192 (9)	0.0026 (10)	−0.0002 (9)	−0.0042 (7)
C2	0.0325 (12)	0.0242 (10)	0.0257 (10)	−0.0049 (10)	0.0011 (9)	−0.0005 (9)
C3	0.0380 (14)	0.0303 (12)	0.0355 (13)	0.0013 (12)	−0.0029 (11)	0.0070 (10)
C4	0.0296 (12)	0.0289 (11)	0.0302 (10)	0.0052 (12)	−0.0059 (10)	0.0043 (9)
C5	0.0342 (14)	0.0237 (11)	0.0346 (11)	−0.0064 (11)	0.0021 (11)	−0.0001 (8)
N1	0.0266 (9)	0.0213 (9)	0.0246 (9)	0.0022 (9)	−0.0012 (7)	−0.0014 (7)
O1	0.0322 (9)	0.0284 (8)	0.0290 (8)	−0.0007 (7)	−0.0092 (7)	0.0032 (6)
O5	0.0456 (12)	0.0447 (11)	0.0398 (9)	0.0131 (9)	−0.0127 (8)	−0.0166 (8)
O3	0.0246 (9)	0.0263 (8)	0.0445 (9)	0.0001 (7)	−0.0038 (8)	0.0019 (7)
O4	0.0416 (10)	0.0427 (11)	0.0411 (11)	−0.0084 (9)	−0.0052 (8)	0.0160 (8)
O2	0.0332 (9)	0.0338 (9)	0.0253 (8)	−0.0011 (8)	−0.0057 (6)	0.0015 (7)
S1	0.0244 (3)	0.0233 (3)	0.0232 (3)	0.0004 (2)	−0.0032 (2)	0.00028 (19)

### Geometric parameters (Å, °)

**Table:**

C1—O1	1.288 (3)	C4—H4B	0.9900
C1—N1	1.303 (3)	C5—N1	1.457 (3)
C1—C2	1.486 (3)	C5—H5A	0.9800
C2—C3	1.532 (3)	C5—H5B	0.9800
C2—H2A	0.9900	C5—H5C	0.9800
C2—H2B	0.9900	O1—H1	0.8400
C3—C4	1.525 (4)	O5—S1	1.4507 (19)
C3—H3A	0.9900	O3—S1	1.5576 (17)
C3—H3B	0.9900	O3—H3	0.8400
C4—N1	1.469 (3)	O4—S1	1.4302 (19)
C4—H4A	0.9900	O2—S1	1.4626 (17)
			
O1—C1—N1	121.0 (2)	C3—C4—H4B	111.1
O1—C1—C2	127.2 (2)	H4A—C4—H4B	109.1
N1—C1—C2	111.9 (2)	N1—C5—H5A	109.5
C1—C2—C3	102.82 (19)	N1—C5—H5B	109.5
C1—C2—H2A	111.2	H5A—C5—H5B	109.5
C3—C2—H2A	111.2	N1—C5—H5C	109.5
C1—C2—H2B	111.2	H5A—C5—H5C	109.5
C3—C2—H2B	111.2	H5B—C5—H5C	109.5
H2A—C2—H2B	109.1	C1—N1—C5	125.3 (2)
C4—C3—C2	104.78 (19)	C1—N1—C4	112.62 (19)
C4—C3—H3A	110.8	C5—N1—C4	122.1 (2)
C2—C3—H3A	110.8	C1—O1—H1	109.5
C4—C3—H3B	110.8	S1—O3—H3	109.5
C2—C3—H3B	110.8	O4—S1—O5	114.49 (13)
H3A—C3—H3B	108.9	O4—S1—O2	112.65 (11)
N1—C4—C3	103.36 (19)	O5—S1—O2	111.19 (11)
N1—C4—H4A	111.1	O4—S1—O3	104.56 (11)
C3—C4—H4A	111.1	O5—S1—O3	106.63 (11)
N1—C4—H4B	111.1	O2—S1—O3	106.60 (10)
			
O1—C1—C2—C3	−168.1 (2)	C2—C1—N1—C5	178.53 (19)
N1—C1—C2—C3	13.3 (3)	O1—C1—N1—C4	−179.01 (19)
C1—C2—C3—C4	−20.2 (2)	C2—C1—N1—C4	−0.3 (3)
C2—C3—C4—N1	20.1 (2)	C3—C4—N1—C1	−13.0 (3)
O1—C1—N1—C5	−0.2 (4)	C3—C4—N1—C5	168.2 (2)

### Hydrogen-bond geometry (Å, °)

**Table:**

*D*—H···*A*	*D*—H	H···*A*	*D*···*A*	*D*—H···*A*
O3—H3···O5^i^	0.84	1.75	2.569 (3)	164
O1—H1···O2	0.84	1.70	2.540 (2)	177
C2—H2A···O5^ii^	0.99	2.45	3.250 (3)	137
C5—H5C···O2^iii^	0.98	2.59	3.488 (3)	152

Symmetry codes: (i) *x*−1/2, −*y*+1/2, −*z*; (ii) *x*+1/2, −*y*+1/2, −*z*; (iii) −*x*+1, *y*+1/2, −*z*+1/2.
